# Distally based sural fasciomusculocutaneous flap for treatment of wounds of the distal third of the leg and ankle with exposed internal hardware

**DOI:** 10.1007/s10195-011-0175-6

**Published:** 2012-01-18

**Authors:** Luca Vaienti, Adriano Di Matteo, Riccardo Gazzola, Pietro Randelli, Jlenia Lonigro

**Affiliations:** 1 Dipartimento di Scienze Medico Chirurgiche, Università degli Studi di Milano, IRCCS Policlinico San Donato, Piazza Malan, 20097 San Donato Milanese, Milan, Italy; 2 Plastic Surgery, Clínica Sant Jordi, Plaça de l’Estació, 12, 08030 Barcelona, Spain

**Keywords:** Internal hardware, Exposure, Infection, Fasciomusculocutaneous sural flap, Lower limb

## Abstract

Soft tissue reconstruction of the distal third of the lower limb with exposure of the internal hardware is a challenging problem with several potential complications, such as exposure of the fracture line, fracture instability and bacterial contamination. The treatment of these lesions usually consists of substitution of the internal hardware with external fixation devices and further flap coverage. We propose a different reconstructive approach, characterized by harvesting a sural fasciomusculocutaneous flap on the exposed internal hardware once a sterile ground has been obtained. Four patients were retrospectively analyzed. Soft tissue reconstruction was achieved in all cases. In one case hardware removal was necessary for complete healing. The sural fasciomusculocutaneous flap is a safe alternative to other pedicled and free flaps. Moreover, it allows direct coverage of internal fixators, thus completing the reconstruction in less time. This flap fits best to the morphology of the wound and internal hardware, leaving the main vascular trunk of the leg intact and at the same time providing a reliable vascular supply.

## Introduction

Soft tissue necrosis after internal fixation with the conventional plate-screw system is quite common. In subjects with fractures treated with the locking compression plate system, Namazi and Mozaffarian [1] observed severe soft tissue damage leading to exposure of internal fixation devices in 23.5% of cases.

The elective treatment of exposed fractures with loss of tissue in the distal third of the leg and the proximal third of the foot classically consists of free or pedicled muscle flap coverage [2]. Nevertheless, the exposed hardware implies an additional problem that is often approached by replacing the internal hardware with external fixators [3] and soft tissue reconstruction in a second surgical period. This strategy requires prolonged healing times (requiring at least two surgeries, one for hardware removal and one for reconstruction), long hospital stays and delayed rehabilitation.

The following series shows that patients with soft tissue infection and internal hardware contamination might benefit from a one-stage debridement and soft tissue reconstruction with a musculocutaneous flap without internal hardware removal.

## Materials and methods

In this study we retrospectively analyzed four patients, affected by exposure of internal hardware and treated with a sural fasciomusculocutaneous flap (Table 1).

**Table 1:** A brief description of the treated patients is given

Case	Patients								
	Age/sex	Weight (kg)	Cause of defect	Defect localization	Defect size (cm)	Systemic pathologies	Smoking	Previous infection	Follow-up (months)
1	72/F	80	Talus-schapoid arthrodesis	Medial malleolus, right foot	7 × 4	Obesity	No	*E*. *coli*, *E*. *faecalis*	24
2	40/M	85	Fixation-plates on fibula and medial malleolus	Medial malleolus, left foot	2 × 1.5	No	40 cigarettes/day	*S*. *aureus*	10
3	53/F	62	Plates and screws on medial malleolus	Distal third of fibula, left foot	4 × 1.5	No	No	*E*. *cloacae*	9
4	74/F	65	Bi-malleolar fracture and fixation with plates and screws	Medial malleolus, right foot	8 × 4	Type II diabetes mellitus	No	*E*. *cloacae*	7

Each patient underwent precise preoperative preparation that included:

- Assessment of proper fracture reduction and correct positioning of the internal hardware.
- Vascularization assessment. If the trauma is suspected to have compromised the main vascular axes of the lower limb, arteriography should be performed.
- Exclusion of osteomyelitis. If blood exams (blood counts, C-reactive protein and erythrocyte sedimentation rate) and radiographs show aspecific signs of osteomyelitis, second level investigations should be considered (e.g., scintigraphy, magnetic resonance imaging, bone cultures).
- Preparation of a sterile ground. Wound cultures were acquired on admission.
  - If negative, debridement and pulsed water washings are performed. In this case the sural fasciomusculocutaneous flap can be harvested at the same time surgically.
  - In case of positive cultures, targeted antibiotic therapy is introduced. In addition, several debridements and cleansing sessions in the operating room are performed until negative cultures are achieved. Bone debridement is not performed in the absence of osteomyelitis.

**Fig. 1:**
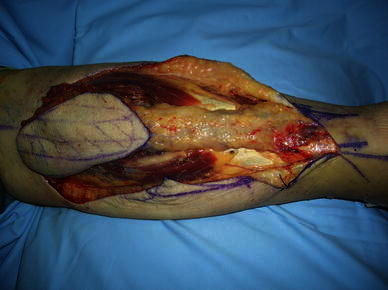
The skin of the cutaneous island is incised to the depth of the muscular fascia, except at the edge above the pedicle where the incision must be extended in depth to the superficial subcutaneous layer. The muscular fascia is then anchored with absorbable stitches to the dermis. The muscular fascia is then raised toward the middle line from both sides. At 2 cm from the gastrocnemius groove, a full-thickness muscular incision is performed parallel to the gastrocnemius groove. The incision ends at the soleus fascia and conserves it. Finally, a cutaneous broken-line incision along the axis of the nerve is made. Cutaneous flaps are lifted, and the sural nerve lies in subcutaneous tissues that are exposed

**Fig. 2:**
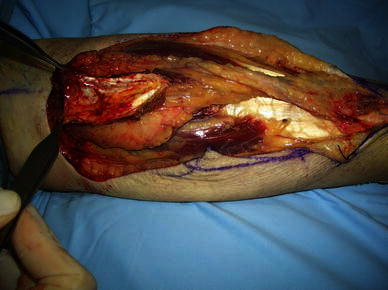
Subcutaneous layers are incised at full-depth to reach the gastrocnemius fascia from both sides. Therefore, a subcutaneous pedicle is manufactured, containing the vascular axis and the sural nerve

Once a sterile ground has been obtained, the sural fasciomusculocutaneous flap operation is performed (Figs. 1, 2).

In one case hyperbaric oxygen therapy (HBOT) was employed preoperatively.

After reconstruction with the sural fasciomusculocutaneous flap, the ankle is immobilized with a plaster splint until complete healing of the skin and soft tissues (usually 15 days in the absence of complications).

Complications occurring in the postoperative period were accurately recorded (e.g., re-exposure of the internal hardware, suture dehiscence, and evidence of local infection).

## Results

Soft tissues reconstruction was achieved in all cases. In one case hardware removal was necessary for complete healing.

### Case 1

**Fig. 3:**
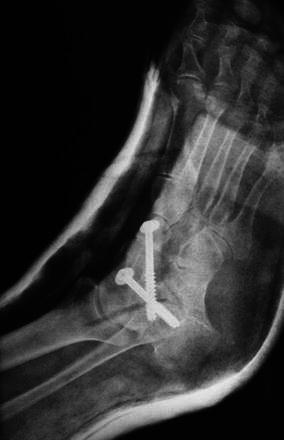
The X-ray shows the talus-scaphoid and talus-calcaneal arthrodesis and the two screws

**Fig. 4:**
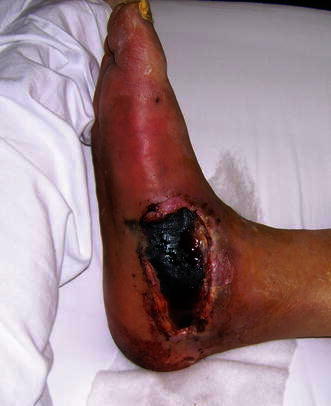
The middle sub-malleolar area of the right foot sustained a soft tissue necrosis with a thick scar

A 72-year-old woman was referred to us after right foot talus-scaphoid and talus-calcaneal arthrodesis through a medial sub-malleolar access (Fig. 3). A full-thickness skin necrosis measuring 7 × 4 cm (Fig. 4) was observed. Infection with *Escherichiacoli* and *Enterococcusfaecalis* was ascertained, while no signs of osteomyelitis were observed.

Arteriography displayed patency of the vascular axis and viability of the posterior tibial artery to the ankle. The size of the defect precluded the use of a local flap, while the depth of the wound and the existence of internal hardware made the sural fasciomusculocutaneous flap preferable to the fasciocutaneous flaps.

**Fig. 5:**
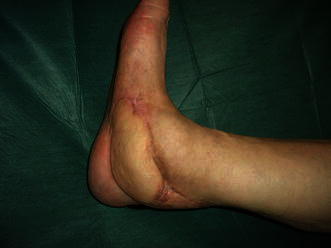
The result 11 months after surgery

Three weeks after surgery a cutaneous fistula appeared on a flap margin. The fistula healed with the removal of the screws 2 months after surgery (Fig. 5).

Full weight bearing was allowed 2 weeks after healing of the fistula.

### Case 2

A 40-year-old man suffering from fibular and medial malleolus fracture of the left leg underwent surgical reduction with plates and screws. Twenty days after the orthopedic surgery, wound dehiscence and exposure of the distal part of the fibular plate were observed. Swabs revealed an infection with *Staphylococcusaureus*. Before any further surgical procedure the patient underwent hyperbaric oxygen therapy, antibiotic therapy and local debridement until cultures were negative. The flap healed uneventfully in 20 days, and full weight bearing was allowed 30 days after surgery. The patient kept smoking during the whole period.

### Case 3

A 53-year-old woman presented with a left foot medial malleolus fracture repaired with plates and screws. Fifteen days after the orthopedic surgery the wound exhibited dehiscence, exposed plates and bacterial infection with *E*. *cloacae*. After antibiotic therapy, pulsed water washings with antiseptics and debridement, a sural fasciomusculocutaneous flap was performed. In the postoperative course a cutaneous dehiscence was observed that healed 20 days after surgery. Ten days later, full weight bearing was allowed.

### Case 4

A 74-year-old woman underwent surgery for reduction and fixation with plates and screws of a bi-malleolar fracture of the right foot. The patient suffered from type II diabetes mellitus and had been on insulin therapy for 20 years. She was also diagnosed with hepatic cirrhosis secondary to chronic hepatitis B and past ascites. Three days after surgery the patient reported extended cutaneous necrosis at the medial malleolus of the right foot. The patient came to our attention 30 days after the first operation with wound dehiscence, exposed plate and infection with *Enterococcuscloacae*. The infection was treated with targeted antibiotic therapy (ciprofloxacin and teicoplanin), debridement and pulsed washings. The exposed hardware was covered with a sural fasciomusculocutaneous flap. Following surgery, a small area of superficial necrosis of the flap occurred. The necrotic area healed in 30 days after debridement and dressings with collagenase. Forty days after surgery, full weight bearing was allowed.

## Discussion

The coverage of internal hardware is a common problem among orthopedic and plastic surgeons. The solution usually consists of removing the internal fixation devices and applying external hardware. However, this strategy displays some drawbacks. In fact, it requires additional surgery in parallel with our approach, thus presenting higher surgical risks, especially in older and unstable patients. Moreover the encumbrance of the external hardware can impair harvesting of a pedicled or a free flap, reducing the reconstructive options in some cases.

According to our experience, the priority should remain the coverage of the damaged area in the shortest of times in order to avoid complications such as exposure of the fracture line, bacterial contamination and fracture instability due to hardware loosening [4].

In our case series, once a sterile ground had been obtained, a sural fasciomiocutaneous flap was harvested and directly applied over the internal fixator.

This strategy is supported by Tan [5] who employed pedicled muscle flaps to cover exposed internal hardware after removal of infection. Mathes [6] stated that internal hardware coverage could be performed also in patients suffering from osteomyelitis.

The required flap should be sufficiently safe, trophic and able to cover the internal hardware filling the death spaces, acting like a “buffer layer.” The sural fasciomusculocutaneous flap can satisfy these requirements, providing a simple and one-stage technique, the vascular reliability of the fasciocutaneous sural flap [7] and the pliancy of a muscular flap to the surfaces of the fixators [8], without compromising the main vascular axes of the leg.

Disadvantages of this flap are the poor aesthetic results at the donor site (especially in those cases that require split-thickness skin grafts), the sacrifice of the sural nerve and the poor pliability (although not limiting) to a curved surface due to the inclusion of the muscle. The possible functional limitation resulting from the partial muscular sacrifice had no clinically relevant consequences.

Muscular flaps (e.g., the soleus muscle flap, extensor digitorum brevis), perforator flaps and free flaps could be employed as alternative to the sural fasciomusculocutaneous flap. Nevertheless, the reliable vascularization, thickness, size, pedicle length and harvesting simplicity make the sural fasciomusculocutaneous flap preferable to cover exposed internal hardware in the distal leg and proximal third of the foot compatibly with the location and size of the defect.

In our case series we employed the sural fasciomusculocutaneous flap on large wounds, achieving full reconstruction of soft tissues and successful coverage of internal hardware in three cases. We observed flap survival in all cases, although suture dehiscence, superficial necrosis and fistulae were observed. Donor site morbidities were not recorded.

Debridements and pulsed water washings are relevant issues for a proper reconstruction. In addition, HBOT could be employed, especially in traumas with extensive damage of soft tissue and impairment of local vascularization (e.g., fractures classified as IIIB or IIIC according to Gustilo) [9]. In fact, HBOT improves tissue oxygenation, thus promoting tissue regeneration and preparing the wound bed's vascular status. HBOT can also be employed postoperatively for flaps suffering from edema, stasis and cyanosis. In our case series, one patient underwent a treatment cycle of 12 sessions [10].

We believe that the sural fasciomusculocutaneous flap is a valuable reconstructive option for wounds of the lower leg and the distal third of the foot with exposure of internal hardware, although larger case series are necessary to precisely define the indications and limits of this versatile reconstructive choice.
